# Internalization and Axonal Transport of the HIV Glycoprotein gp120

**DOI:** 10.1177/1759091414568186

**Published:** 2015-01-28

**Authors:** Sarah Berth, Hector Hugo Caicedo, Tulika Sarma, Gerardo Morfini, Scott T. Brady

**Affiliations:** 1 Department of Anatomy and Cell Biology, University of Illinois at Chicago, IL, USA

**Keywords:** axonal transport, distal sensory polyneuropathy, gp120, HIV, lipid rafts

## Abstract

The HIV glycoprotein gp120, a neurotoxic HIV glycoprotein that is overproduced and shed by HIV-infected macrophages, is associated with neurological complications of HIV such as distal sensory polyneuropathy, but interactions of gp120 in the peripheral nervous system remain to be characterized. Here, we demonstrate internalization of extracellular gp120 in a manner partially independent of binding to its coreceptor CXCR4 by F11 neuroblastoma cells and cultured dorsal root ganglion neurons. Immunocytochemical and pharmacological experiments indicate that gp120 does not undergo trafficking through the endolysosomal pathway. Instead, gp120 is mainly internalized through lipid rafts in a cholesterol-dependent manner, with a minor fraction being internalized by fluid phase pinocytosis. Experiments using compartmentalized microfluidic chambers further indicate that, after internalization, endocytosed gp120 selectively undergoes retrograde but not anterograde axonal transport from axons to neuronal cell bodies. Collectively, these studies illuminate mechanisms of gp120 internalization and axonal transport in peripheral nervous system neurons, providing a novel framework for mechanisms for gp120 neurotoxicity.

## Introduction

The HIV glycoprotein gp120 is a neurotoxic viral protein that is overproduced and shed during HIV infection (Schneider et al., 1986). Infected macrophages are thought to damage sensory neurons indirectly by producing neurotoxic factors including cytokines, excitatory amino acids, and shed viral proteins (Kaul et al., 2005). Several lines of evidence link gp120 to neurological complications of HIV, such as distal sensory polyneuropathy (DSP; Ellis et al., 2010; Evans et al., 2011; Anziska et al., 2012). Notably, transgenic expression of gp120 in mice suffices to produce dying back neuronal pathology (Toggas et al., 1994; Michaud et al., 2001). Additionally, infected macrophages infiltrate dorsal root ganglia (DRGs), promoting a higher local concentration of gp120 around the DRG neurons (Laast et al., 2011), and administration of recombinant gp120 to rodents causes pain hypersensitivity, a common DSP symptom (Milligan et al., 2000; Herzberg and Sagen, 2001). Gp120 can directly bind to its coreceptors CXCR4 and CCR5 on the surface of DRG neurons (Apostolski et al., 1993; Oh et al., 2001), with CXCR4 being more clearly linked to neurotoxicity than CCR5 (Kaul et al., 2005). Consequently, T-tropic strains of gp120 that bind to CXCR4, as opposed to M-tropic strains of gp120 that bind to CCR5, are more commonly utilized to study gp120 neurotoxicity.

Recently, gp120 was reported to be internalized by neurons of the central nervous system (CNS; Bachis et al., 2003, 2006), raising the possibility that intracellular gp120 might also be harmful to neurons. However, pathways by which gp120 may be internalized have not been identified. An understanding of how gp120 enters neurons and how sensory neurons respond to viral protein entry could lead to an enhanced knowledge of mechanisms underlying gp120 neurotoxicity. In this context, identifying pathways for gp120 internalization would aid in development of novel therapeutic strategies either by preventing gp120 entry into sensory neurons or blocking downstream signaling pathways.

Major pathways that are candidates for gp120 internalization include the common endosomal pathway, which utilizes clathrin-mediated endocytosis; fluid phase pinocytosis in which endosomes are nonspecifically taken up by the cell along with extracellular fluid; and lipid raft internalization. Lipid rafts are cholesterol-enriched, discrete microdomains within the plasma membrane that act as signal transduction platforms and mediate clathrin-independent internalization. Several viruses utilize lipid rafts to mediate entry into cells (Chazal and Gerlier, 2003; Suzuki and Suzuki, 2006), including HIV (Chazal and Gerlier, 2003). Accordingly, gp120 clusters with its coreceptors in lipid rafts to allow virus internalization (Manes et al., 2000; Liao et al., 2001; Popik et al., 2002), making lipid rafts likely sites for gp120 entry.

Neurons of the peripheral nervous system (PNS) are remarkably polarized, with axons reaching up to a meter or more in length in some cases. This unique cellular architecture renders these cells highly dependent on microtubule motor-dependent fast axonal transport mechanisms for trafficking of proteins and cellular components within axons (Morfini et al., 2009). Certain viruses, including herpes simplex virus and adenoviruses, have the capability to hijack specific components of the host’s fast axonal transport machinery (i.e., molecular motors and vesicle-associated proteins), allowing their intracellular movement (Berth et al., 2009). Transport of gp120 itself has been reported in the CNS but not in PNS axons (Ahmed et al., 2009).

The aims of this study were to determine whether gp120 is subject to internalization by PNS sensory neurons, to identify specific uptake pathways involved, and to evaluate whether gp120 is transported along axons. Our findings indicate that gp120 is mainly internalized through lipid rafts, with a minor fraction internalized through fluid phase pinocytosis. Additionally, gp120 was retrogradely transported from axons to the cell bodies in a time frame indicating fast axonal transport. The demonstration of intracellular internalization for gp120 in DRG neurons raises the possibility of intracellular neurotoxic effects of gp120.

## Materials and Methods

### Cell Culture

F11 cells (a generous gift from Dr. Richard Miller) were grown in high glucose DMEM media (Invitrogen), supplemented with 10% fetal bovine serum (FBS), 1% glutamax, and 10,000 U/ml penicillin-streptomycin. Six-channel µ-slides from Ibidi (#80606) were coated with 0.1 µg/ml poly-L-lysine (Sigma) and then rinsed 4 times (30 min each) with autoclaved, deionized water. Then, 300 cells were added to each channel and maintained at 37℃ in 5% CO_2_ and 95% O_2_. To differentiate F11 cells, the amount of FBS was first reduced to 5% for 24 hr, and cells were treated with 0.5 mM dibutryl-cAMP (Sigma) in media with 0.5% FBS for 4 days.

### Antibodies and Reagents

The following antibodies were used: DM1A (Sigma), CXCR4 (Abcam #ab2074), EEA1 (Cell Signaling #3288), and LAMP-2 (Thermo Scientific #PA1-655). Recombinant gp120 IIIB and fluorescein-conjugated recombinant gp120 IIIB were purchased from Immunodiagnostics (#1001 and #1001-F, respectively). Gp120 BaL and AMD3100 (AMD) were obtained from the National Institutes of Health AIDS Research and Reference Reagent Program. Rhodamine-dextran (10,000 MW), Alexa Fluor 594-cholera toxin B, and Alexa Fluor-transferrin were purchased from Invitrogen. The HRP-conjugated secondary goat anti-rabbit antibody was purchased from Jackson. Methyl-β-cyclodextrin (CD) was purchased from Sigma.

### Cell Lysates

F11 cells were differentiated in 100 mm Petri dishes. After 4 days of 0.5 mM dibutryl-cAMP (Sigma) treatment, cells were scraped and collected in 700 µL lysis buffer (1% sodium dodecyl sulfate [SDS] in phosphate-buffered saline [PBS], pH 7.4) and sonicated for two short, 3-s bursts each. Cells were spun at 14,000 rpm at 4℃ in a Beckman tabletop centrifuge. Protein concentration of the clarified supernatants was determined using a bicinchoninic acid assay (BCA assay) kit (Pierce). Sample buffer was added to the clarified supernatants, and the samples were placed in the −20℃ freezer prior to analysis via SDS-PAGE.

### Immunoblots

Protein samples were loaded onto a 4% to 12% bis/tris gradient gel (Invitrogen). Proteins from the gels were transferred (at 4℃) to Immobilon-P transfer membranes (PVDF, Millipore) at 0.4 Amps for 2 hr in 1× Towbin buffer. Membranes were blocked at room temperature for 120 min with 5% milk in Tris-buffered saline (TBS). Primary antibody (Abcam #2074) in 1% bovine serum albumin (BSA) was added overnight at 4℃ with gentle rocking. The primary antibody was washed 3 × 10 min with TBST (0.1% Tween), and the secondary antibody was added for 1 hr at 4℃ with gentle rocking. Membranes were again washed 3 × 10 min and visualized with ECL (Millipore) and exposed on film (Kodak) for HRP secondary.

### Treatment of Cells

Differentiated F11 cells were treated with fluorescein-conjugated gp120 IIIB for several time points. For heat inactivation, gp120 IIIB was boiled for 1 hr prior to treating F11 cells. For experiments in Figure 2, F11 cells were preincubated with 2 µM AMD for 1 hr and then cotreated with 2 µM AMD and 70 nM (8.3 µg/ml) fluorescein-gp120 for a 2-hr time course. For CD pretreatment experiments, F11 cells were incubated with 5 mM CD for 20 min, washed 3 times with media, and then incubated with 70 nM fluorescein-gp120 for a 2-hr time course. To examine the effect of CD pretreatment on transferrin internalization, differentiated F11 cells were treated with either 5 mM CD or washed with media for 20 min at 37℃, incubated with 50 µg/ml transferrin on ice for 15 min, and then washed and incubated at 37℃ for 15 min. For colocalization analysis with dextran, F11 cells were cotreated with 70 nM gp120 and 200 µg/ml dextran for a 2-hr time course. To examine colocalization between gp120 and cholera toxin B, differentiated F11 cells were first incubated with 500 ng/ml cholera toxin B on ice for 15 min, washed, and then incubated with 70 nM fluorescein-gp120 at 37℃ for a 2-hr time course. Cells were then fixed with 4% paraformaldehyde and 0.01% glutaraldehyde in PBS for 10 min and quenched with 50 mM ammonium chloride in PBS for 5 min. Mounting media with the nuclear dye DAPI (Vectashield) was dropped into wells.

### Immunocytochemistry

All steps were carried at room temperature, unless indicated. DM1A (anti-alpha tubulin) antibody was used to visualize F11 cells and their neurites, or DRG neurons and their axons. For immunostaining with DM1A, fixed F11 cells were permeabilized with 0.1% triton-X-100 for 10 min and then blocked with 5% fat-free milk in PBS for 1 hr. Cells were incubated with DM1A antibody at room temperature for 1 hr. For immunostaining with CXCR4 antibody, fixed F11 cells were permeabilized with 0.1% triton-X-100 for 10 min and then blocked with 1% BSA in PBS for 1 hr. Cells were incubated with CXCR4 antibody at room temperature for 1 hr. For immunostaining using EEA1 and LAMP2 antibodies, F11 cells were permeabilized with 0.25% triton-X-100 in PBS for 1 hr and blocked with 5% normal goat serum (Santa Cruz). Cells were incubated with primary antibodies for 1 hr, washed, and incubated with Alexa Fluor 594-conjugated secondary antibody for 1 hr at room temperature. Mounting media with DAPI (Vectashield) was dropped into wells.

### Fluorescence Imaging and Quantitation

Cells were imaged using a Zeiss LSM 510 Meta laser scanning confocal microscope with a 100× objective. One-micrometer-thick z-stacks were obtained, and at least 10 cells per experimental condition were analyzed for each time point. Z-stacks excluded the slices in which the upper and lower boundaries of the cells were in focus, to ensure that the z-stacks were only composed of intracellular slices. For the CXCR4 image, a 40× objective was used to show multiple cells.

Images were deconvolved and analyzed in three dimensions using Volocity software (Perkin Elmer). Three-dimensional reconstructions and movies were created in Volocity (Supplemental Figures 1 to 3). For internalization assays, the average fluorescence units were calculated for each region of interest (ROI) obtained from each cell traced. For colocalization analysis, the Pearson’s correlation coefficient, which calculates the correlation between intensities of different channels, was obtained for each cell. To provide quantitative colocalization of immunofluorescent signals, colocalization coefficients M1 and M2 were obtained for the green and red channels respectively which calculate the fraction of each channel that overlapped with the other channel (Manders et al., 1993). For quantitation of gp120 internalization, Student’s *t* test and Spearman’s correlation were used for statistical analysis. Analysis of variance (ANOVA) was utilized to compare amounts of gp120 internalization between treatments of gp120 alone, heat inactivated gp120, and AMD pretreatments. Student’s *t* test was used to examine decreases in gp120 internalization after CD treatment for 30 min, 1 hr, and 2 hr time points. Quantitative data were expressed as mean ± *SEM*, and significance was determined at *p* < .05.

**Figure 1.**
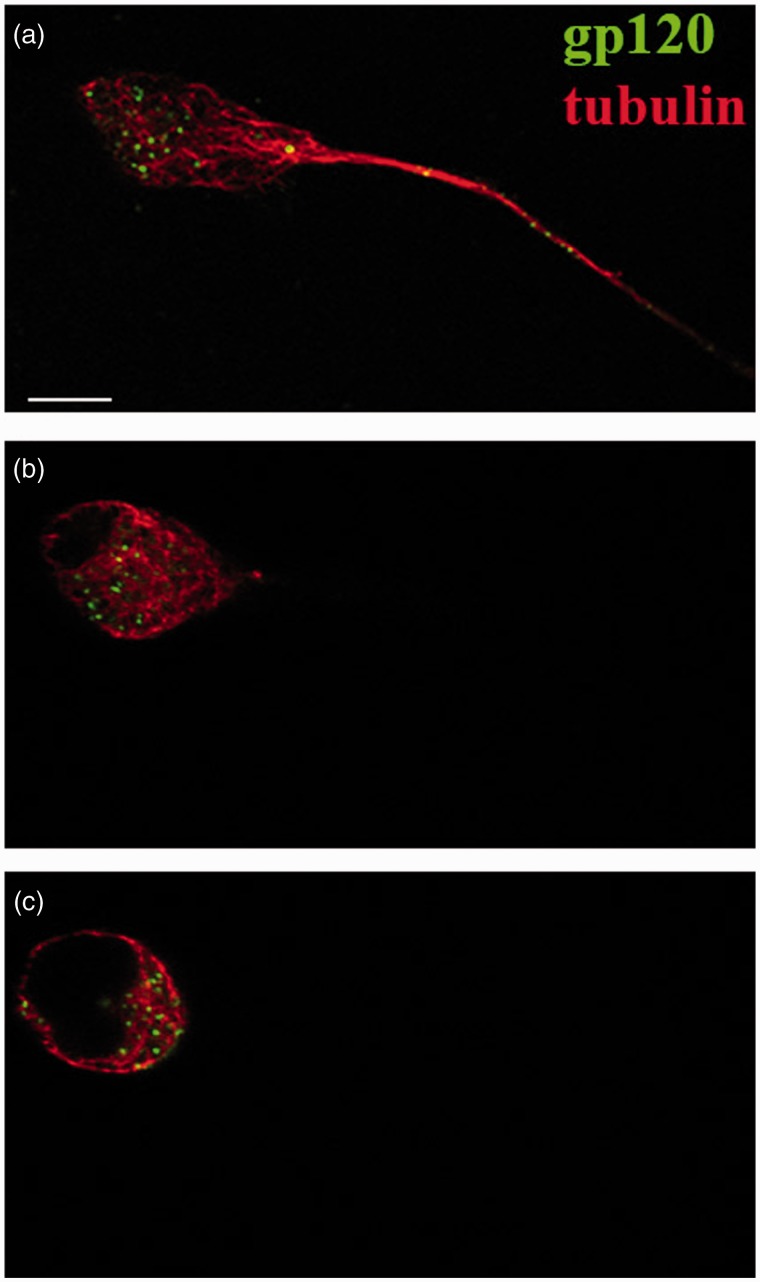
Gp120 internalization by F11 cells. Representative z-stack from confocal microscopic analysis of F11 cells treated with fluorescein-gp120 for 2 hr. Panels show the bottom (a), middle (b), and top of an F11 cell (c). Fluorescein-gp120 (green) accumulated in the perinuclear region of the middle slices of the cell (b), demonstrating that gp120 was internalized. The morphology of cells was revealed using an anti-tubulin antibody (red). Scale bar: 10 µm.

**Figure 2.**
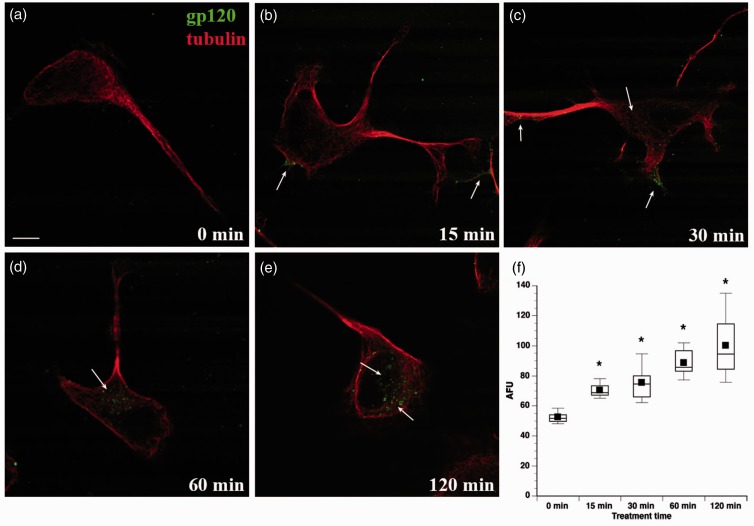
Time course of gp120 internalization by F11 cells. F11 cells were treated with fluorescein-gp120 (green) for 0 (a), 15 (b), 30 (c), 60 (d), and 120 min (e). Arrows in (b) and (c) point to fluorescein-gp120 accumulated at the cell membrane and neuritic processes. By 30 min, gp120 begins to accumulate in perinuclear areas of the cell (c), with the amount of perinuclear gp120 increasing at later time points (d) and (e). Scale bar: 10 µm. The morphology of cells was revealed using an anti-tubulin antibody (red). (f) Average gp120-derived fluorescence was calculated for 10 or more cells and box plots generated for each time point (see Materials and Methods section). Intracellular gp120 fluorescence significantly increased by 15 min of treatment (*p < .0001), and continued to increase for the duration of the treatment, showing signs of saturation at the 2-hr time point.

**Figure 3.**
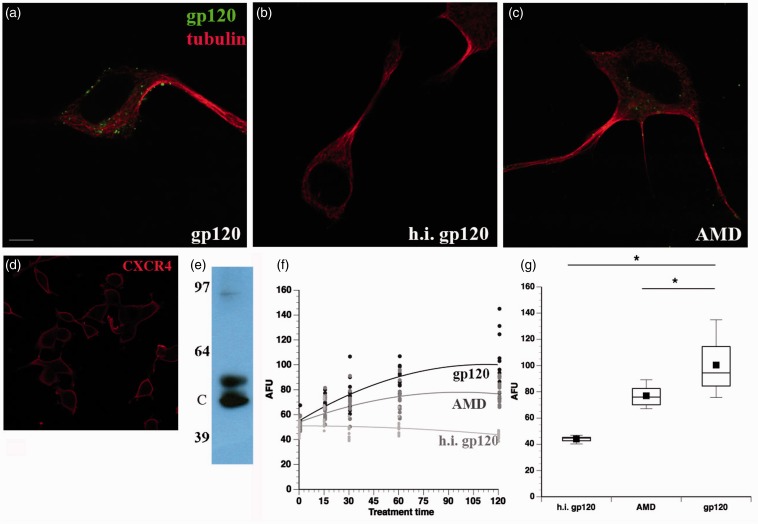
Gp120 internalization is abolished by heat inactivation and reduced by AMD3100. F11 cells were treated with (a) fluorescein-gp120, (b) heat-inactivated (h.i.) gp120, or (c) pretreated with 2 µm AMD3100 (AMD; an inhibitor of gp120 binding to CXCR4) for 1 hr before the addition of fluorescein-gp120. Heat inactivation prevented internalization of fluorescein-gp120 by F11 cells, demonstrating that internalization is specific to the native protein conformation of gp120. Also, AMD3100 treatment reduced gp120 internalization, demonstrating that internalization of gp120 is partially dependent on binding to CXCR4, a normal biological activity. However, the partial reduction of internalization indicates that gp120 can also be internalized through a mechanism independent of CXCR4. The morphology of cells was revealed using an anti-tubulin antibody (red). (d) F11 cells immunostained with a CXCR4 antibody demonstrate the presence of CXCR4 on the surface of the cells. Scale bar: 10 mm. (e) Immunoblot confirms the presence of CXCR4 in F11 cells. C indicates the expected MW of CXCR4. (f) Average gp120-derived fluorescence was calculated for 10 or more cells and box plots generated for each time point (see Materials and Methods section). Each circle denotes an individual measurement. The time course of heat-inactivated gp120 (light gray) is compared with the time course of gp120 pretreated with AM3100 (dark gray) and fluorescein-gp120 treatment (black). (g) Box plots show quantitation of gp120 internalization after 2 hr of treatment. Both AMD3100 pretreatment and heat inactivation of gp120 significantly reduced the amount of gp120 internalization to varying extents (e). *p < 0.01.

### Microfluidic Devices

Custom microfluidic chambers were produced to compartmentalize axonal terminal and somatodendritic compartments (ATC and SDC, respectively) of cultured neurons. Nanofabricated microchannels separating these compartments are small enough (length of 900 µm and a width of 8 µm) that only axons can grow through. Fluidic isolation of a compartment is maintained by volume differences of 200 µl, as well as through a perpendicular central channel 23 mm long and 50 µm wide, so that the pressure gradient overcomes diffusion. Microfluidic chambers were fabricated using photolithography and replica molding techniques. Briefly, patterned layers of PDMS were exposed to plasma generated by a laboratory corona treater (BD-20AC; Electro-Technic Products, Inc., Chicago, IL), as were glass coverslips. The plasma-treated surfaces were then immediately placed in conformal contact, and incubation of the whole setup at 80℃ overnight resulted in irreversible bonding. ATC and SDC were loaded with 70% ethanol for 30 min, rinsed with autoclaved water 4 times, and if needed, treated with 0.1% tritonX-100 in PBS to remove bubbles from the microchannels. After this, devices were washed with autoclaved water 3 times for 5 min each.

Microfluidic devices were incubated overnight with 0.5 mg/ml poly-L-lysine (Sigma) in 0.1 M borate buffer pH 8.5 and then washed with autoclaved water 4 times for 30 min each. Devices were incubated overnight with 10 µg/ml laminin (Invitrogen) in Neurobasal media (Invitrogen) at 37℃ prior to plating the DRG neurons.

### Primary DRG Neuron Cell Culture

All experiments involving animals were conducted according to protocols approved by the Institutional Animal Care and Use Committee at University of Illinois at Chicago. E15 DRG neurons were dissected from embryos obtained from timed pregnant Sprague-Dawley rats, as described in Hall et al. (2006), and placed into ice-cold 1× Hank’s balanced salt solution. Trypsin was added to 0.25%, and the cells were incubated at 37℃. Trypsin was inactivated by adding 10% FBS, and cells were collected by centrifugation (600 × g for 6 min). Cells were resuspended in serum-free Neurobasal media (Invitrogen) supplemented with 2% B27 (Invitrogen), 1% Glutamax (Invitrogen), 0.5% penicillin/streptomycin (10,000 U/ml each, Invitrogen), and 2.5 S nerve growth factor (NGF; 20 ng/ml, Invitrogen). Cells (90,000) were added to the SDC of the prepared microfluidic devices (30,000 cells per SDC compartment), and microfluidic flow was from SDC to ATC. Within the ATC, 2.5 S NGF was added at 50 ng/ml concentration, to maintain an NGF gradient that attracts DRG axons into the ATCs. Half of the cell media was replaced with fresh media every other day. 5-Fluoro-2′-dexyuridine (FudR; 10 µm) was added to the cell media every other time the media was replaced to eliminate nonneuronal cells.

## Results

### Time Course of gp120 Internalization by F11 Cells

The neurotoxicity of gp120 was commonly thought to result from binding to its coreceptor CXCR4 at the plasma membrane and abnormal activation of signaling pathways (Kaul et al., 2005), but internalization of gp120 by some neurons of the CNS was recently demonstrated (Bachis et al., 2003, 2006). However, the pathway for this internalization and gp120 uptake by sensory neurons, the main neuronal type affected in DSP, has not been established. Examining gp120 internalization by sensory neurons could illuminate mechanisms for sensory neuron-specific responses to gp120, providing novel insights into DSP pathogenesis. To address this issue, we evaluated gp120 internalization in F11 cells differentiated with dibutryl-cAMP, which are rat DRG neurons hybridized with mouse neuroblastoma cells (Ghil et al., 2000). F11 cells were chosen for these experiments due to their similarity to DRG neurons. Their ability to proliferate and differentiate avoids the heterogeneity of neuronal cell type characteristic of DRG primary cultures (Francel et al., 1987; Shastry et al., 2001). This is important to avoid any indirect effects of gp120 treatment through its interactions with nonneuronal cells. To follow uptake and internalization, fluorescein-conjugated gp120 IIIB (70 nM) was added to the culture media. Z-stack images obtained by confocal microscopy demonstrated accumulation of gp120 within cells after 2 hr of incubation (Figure 1(a) to (c), Supplemental Figures 1 to 3). A time-course study (Figure 2(a) to (e)) further indicated that internalization of gp120 by F11 cells was detectable as early as 15 min (Figure 2(b)) and continued to increase for more than 2 hr (Figure 2(e)). The gp120 was first seen to be localized along neuritic processes (not shown) and at cell bodies, with accumulation in the perinuclear region of the cell bodies at later time points, raising the possibility that this protein may undergo retrograde axonal transport (see later). A Student’s two-tailed *t* test revealed that the amount of gp120-derived average fluorescence was significantly increased after 15 min of treatment (*p* < .0001) and continued to increase for the duration of the treatment (Figure 2(f)). A Spearman’s correlation analysis confirmed that gp120 internalization increased over time (*p* < .001).

### Gp120 Internalization Is Specific and Partially Dependent on Binding to CXCR4

To evaluate whether gp120 internalization was specific to its biological activity, fluorescein-gp120 was heat inactivated by boiling for 1 hr before addition to F11 cells. Internalization of gp120 (Figure 3(a)) over the course of 2 hr was completely abrogated by heat inactivation of gp120 (Figure 3(b)), demonstrating that internalization of gp120 by F11 cells required native protein conformation. Because T-tropic gp120 binds to its coreceptor CXCR4 on DRG neurons (Apostolski et al., 1993; Oh et al., 2001), the necessity of this binding for internalization was evaluated. Immunocytochemistry experiments (Figure 3(d)) and immunoblots (Figure 3(e)) confirmed the presence of CXCR4 in F11 cells. F11 cells were pretreated for an hour with 2 µM AMD, a small molecule inhibitor of gp120 binding to CXCR4 (De Clercq et al., 1994; Donzella et al., 1998), and then a time course experiment was performed cotreating cells with fluorescein-gp120 and AMD. Significantly, treatment of F11 cells with AMD markedly reduced the amount of internalized gp120 (Figure 3(c)) compared with untreated cells (Figure 3(a)), but not to the same extent as heat-inactivated fluorescein-gp120 Figure 3(b)). This concentration of AMD was sufficient to abolish signaling through the CXCR4 receptor (data not shown).

A one-way ANOVA was performed to determine differences in the amounts of gp120 internalized between the gp120 alone group, the heat-inactivated gp120 group, and the AMD treated group (Figure 3(e) and (f)). The groups were found to be significantly different (*p* < .0001). Further, all experimental groups were significantly different from each other (*p* < .0001 for each comparison) using a Tukey–Kramer test. These data indicate that gp120 binding to CXCR4 is necessary for some, but not all of the gp120 internalized by F11 cells.

### Mechanisms of gp120 Internalization

**Figure 4.**
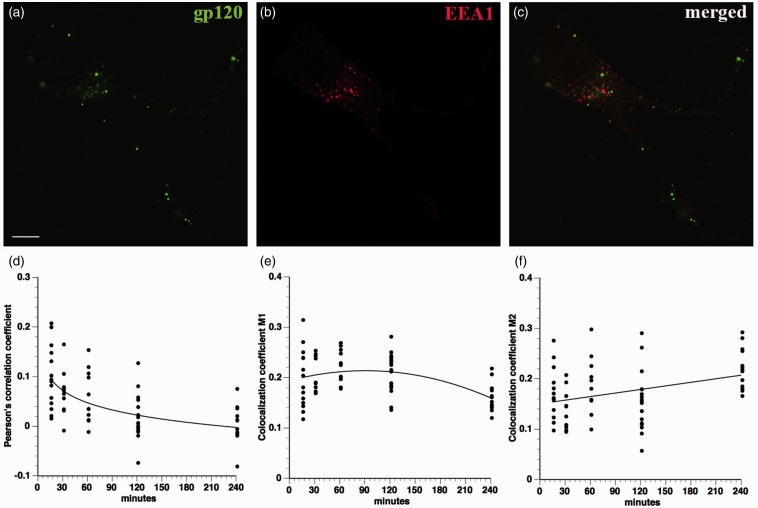
Gp120 does not colocalize with EEA1. A time course of fluorescein-gp120 internalization by F11 cells was performed, and cells were stained with an antibody for EEA1, a marker of early endosomes within the common endosomal pathway. Representative images of an F11 cell treated for 2 hr show fluorescein-gp120 (a, green), EEA1 immunoreactivity (b, red), and both gp120 and EEA1 (c) Scale bar: 10 µm. Z-stacks were taken of at least 10 cells per time point on a laser scanning confocal microscope, and images were deconvolved and analyzed for colocalization in Volocity. The Pearson’s correlation coefficient (d), colocalization coefficient M1 (green channel; e) and colocalization coefficient M2 (red channel; f) all show very low values, consistent with lack of co-localization for fluorescein-gp120 and EEA1. This indicates that gp120 is not significantly internalized through the common endosomal pathway.

**Figure 5.**
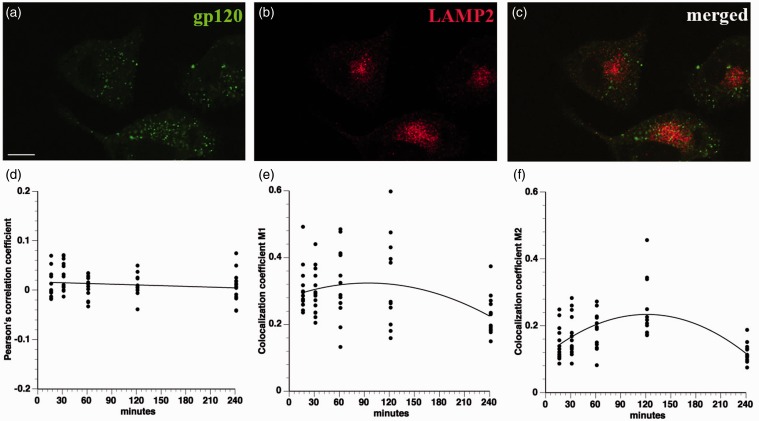
Gp120 does not colocalize with LAMP2. A time course of fluorescein-gp120 (green) internalization by F11 cells was performed, and cells were stained with an antibody for LAMP2 (red). The scale bar denotes 10 µm. A representative image of a F11 cell treated for 2 hr is shown in (a) to (c). Note the lack of colocalization in (c) between green and red channels. Z-stacks were taken of at least 10 cells per time point on a laser scanning confocal microscope, and images were deconvolved and analyzed for colocalization in Volocity. The Pearson’s correlation coefficient (d), colocalization coefficient M1 (green channel; e), and colocalization coefficient M2 (red channel; f) all show very low values, demonstrating that fluorescein-gp120 does not colocalize with LAMP2. Because LAMP2 is a marker of lysosomes in the common endosomal pathway, this indicates that gp120 is not internalized through the common endosomal pathway.

The highly punctate intracellular staining of fluorescein-conjugated gp120 (Figure 1) suggested internalization through an endocytic mechanism. To evaluate this possibility, F11 cells were treated with fluorescein-conjugated gp120 for a time course more than 4 hr and then stained with antibodies recognizing markers of the early (EEA1) and late (LAMP2) endosomal/lysosomal compartments. Colocalization of internalized gp120 with these markers was evaluated using the Pearson’s correlation coefficient along with the colocalization coefficients M1 and M2 (see Fluorescence Imaging and Quantitation section). Significantly, gp120 failed to colocalize with either EEA1 (Figure 4) or LAMP2 (Figure 5) throughout the entire time course of the experiments, ruling out internalization through the common endolysosomal pathway.

**Figure 6.**
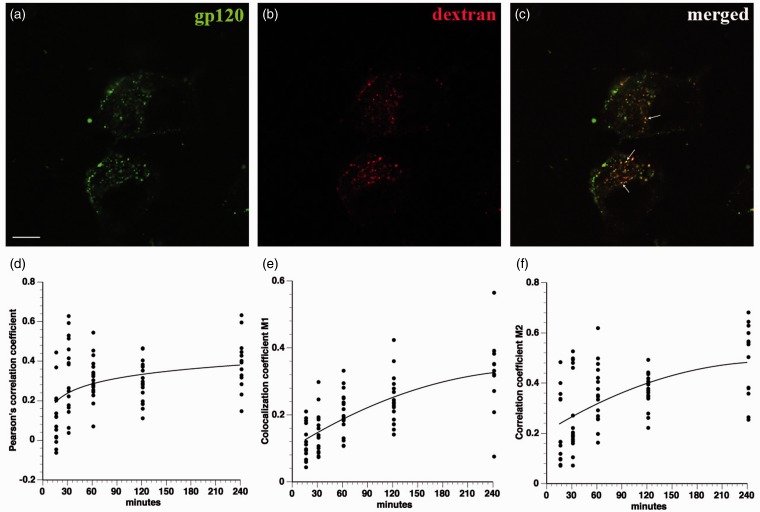
Gp120 partially colocalizes with dextran. F11 cells were cotreated with fluorescein-gp120 (green) and rhodamine-dextran (red), and a time course of internalization was performed. The scale bar denotes 10 µm. A representative image of a F11 cell treated for 2 hr is shown in (a) to (c). Note the partial colocalization in (c) between green and red channels. Arrows in (c) point to colocalized puncta. Z-stacks were taken of at least 10 cells per time point on a laser scanning confocal microscope, and images were deconvolved and analyzed for colocalization in Volocity. The Pearson’s correlation coefficient (d), colocalization coefficient M1 (green channel; e), and colocalization coefficient M2 (red channel; f) all show values indicative of partial colocalization between gp120 and dextran. Because dextran is a marker of fluid phase pinocytosis, this demonstrates that a component of gp120 is internalized through fluid phase pinocytosis.

Next, fluid phase pinocytosis was assessed as a mechanism for gp120 internalization. To this end, differentiated F11 cells were simultaneously treated with fluorescein-gp120 and rhodamine-conjugated dextran (MW 10,000), a well-known marker of fluid phase pinocytosis (Frost et al., 2009). Partial colocalization between gp120 and dextran was observed (Figure 6), suggesting that at least a fraction of extracellular gp120 is internalized into F11 cells through fluid phase pinocytosis.

**Figure 7.**
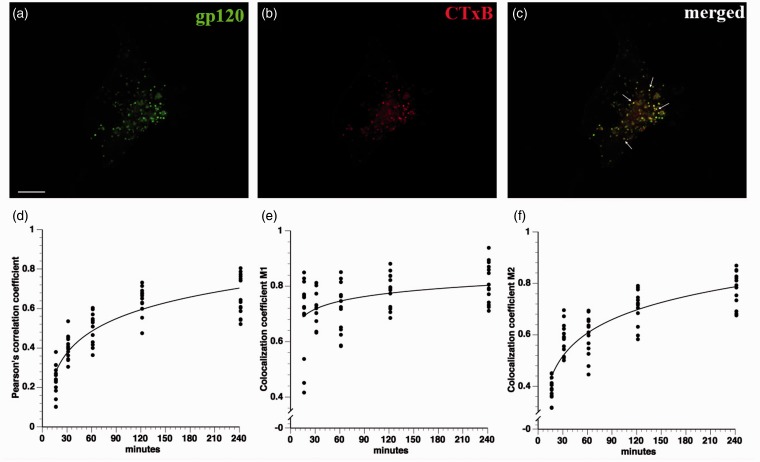
Gp120 substantially colocalizes with cholera toxin B. F11 cells were cotreated with fluorescein-gp120 (green) and Alexa Fluor 594-cholera toxin B (CTxB, red), and a time course of internalization was performed. The scale bar denotes 10 µm. A representative image of a F11 cell treated for 2 hr is shown in (a) to (c). Note the colocalization in (c) between green and red channels. Arrows in (c) point to colocalized puncta. Z-stacks were taken of at least 10 cells per time point on a laser scanning confocal microscope, and images were deconvolved and analyzed for colocalization in Volocity. The Pearson’s correlation coefficient (d), colocalization coefficient M1 (green channel; e), and colocalization coefficient M2 (red channel; f) all show values indicative of colocalization between gp120 and cholera toxin B, especially by 2 hr treatment. Because cholera toxin B is a marker of internalization through lipid rafts, this indicates that gp120 is also internalized through lipid rafts.

**Figure 8.**
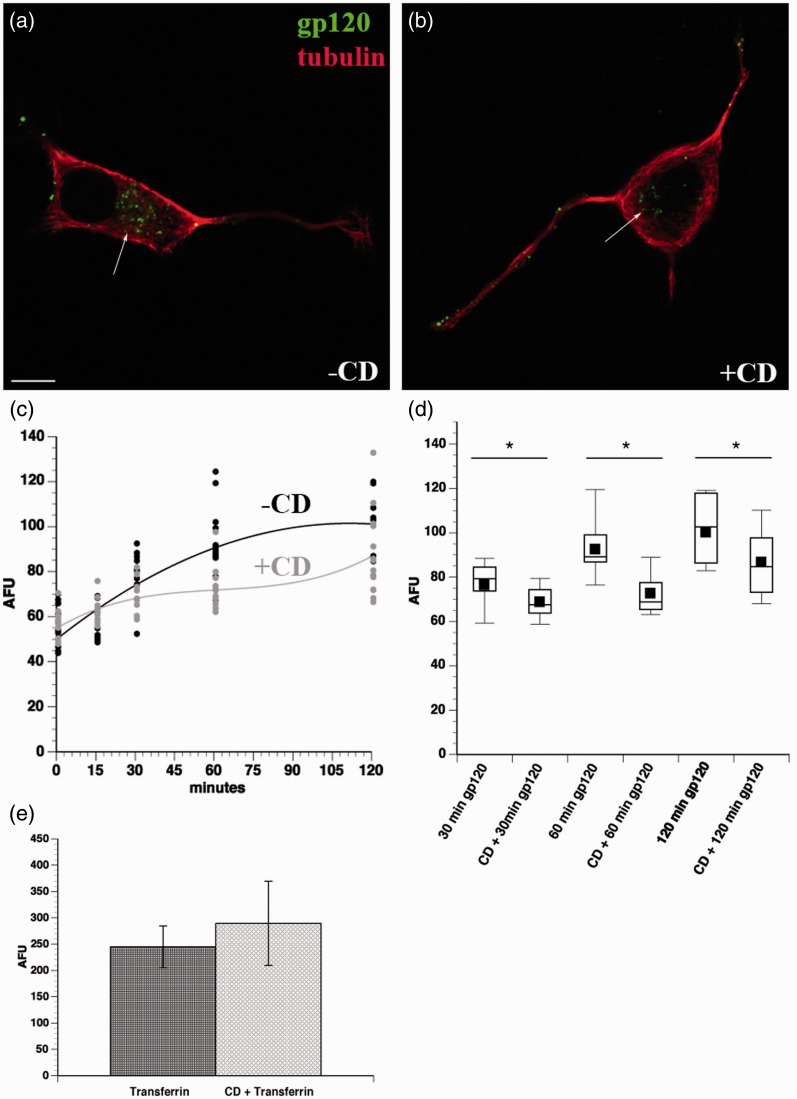
Cyclodextrin treatment reduces internalization of gp120. F11 cells were pretreated with 5 mM β-methyl-cyclodextrin (CD) for 20 min to disrupt lipid rafts, and then a time course of internalization of fluorescein-gp120 was performed. Scale bar: 10 µm. Representative images of cells treated with fluorescein-gp120 for 2 hr without CD (a) or with CD pretreatment are shown (b). The morphology of cells was revealed using an anti-tubulin antibody (red). Note the reduced amount of internalized gp120 in CD-treated cells (b), compared with untreated ones (a). Average gp120-derived fluorescence was calculated for 10 or more cells. As shown in (c), pretreatment with 5 mM cyclodextrin for 20 min reduced the amount of internalized gp120 (gray line), compared with control cells (black line). Each measurement is plotted in (c), with gray and black dots corresponding to pretreatment with CD or no pretreatment, respectively. (d) Box plots demonstrate that internalization of gp120 was significantly reduced at the 30, 60, and 120 min time points, consistent with internalization of gp120 through lipid rafts. *p < .05. (e) Pretreatment of differentiated F11 cells with 5 mM cyclodextrin for 20 min did not significantly affect internalization of transferrin through the clathrin-mediated pathway (e).

Because gp120 did not enter the cell through the common endolysosomal pathway and fluid phase pinocytosis only accounted for a minor fraction of total gp120 internalized, we considered that gp120 might also be internalized through lipid raft-mediated endocytosis, another non-clathrin-mediated process (Lajoie and Nabi, 2010). Gp120 and its receptors are known to cluster in lipid rafts (Popik et al., 2002), and this clustering is critical for HIV infection (Kamiyama et al., 2009). Consistent with this possibility, significant colocalization was observed between internalized fluorescein-gp120 and cholera toxin B (Figure 7). To further evaluate the role of lipid rafts for gp120 internalization, differentiated F11 cells were treated with 5 mM CD for 20 min to deplete cholesterol and inhibit lipid raft internalization. Next fluorescein-gp120 was added to media, and gp120 internalization quantified. Comparing untreated cells (Figure 8(a)) with F11 cells pretreated with CD (Figure 8(b)) indicated that CD pretreatment significantly decreased the amount of internalized gp120 at 30, 60, and 120 min time points (Figure 8(b) to (d)), and the differences were significant at *p* < .05 using a Student’s two-tailed *t* test (*), a finding consistent with results from colocalization experiments in Figure 7. In concurrent experiments, treatment of F11 cells with CD did not decrease the amount of internalized transferrin (Figure 8(e)), demonstrating that the inhibitory effect of CD on gp120 internalization was specific to lipid raft-mediated, but not clathrin-mediated, endocytosis. Taken together, these experiments indicated that lipid raft-mediated endocytosis represents a major pathway for gp120 internalization by sensory neurons.

### Recombinant gp120 Is Transported Retrogradely, but Not Anterogradely, Along DRG Axons

A number of viruses and viral proteins have the capability to highjack various components of the host’s axonal transport machinery and be transported to different intracellular compartments (Berth et al., 2009). Having established internalization of gp120 by F11 cells, we examined internalization and axonal transport of gp120 in cultured DRG neurons using compartmentalized microfluidic devices, which allow for isolation of SDC and ATC. All axons have the same polarity and are in register, so directionality of transport can be evaluated in the axons. The diameter of nanofabricated microgrooves that separate the SDC and ATC prevents dendrites or cell bodies from growing into the ATC. Primary DRG neurons bearing differentiated axons are more suited for these devices than F11 cells, so rat primary DRG neurons were utilized for these experiments to directly evaluate axonal trafficking.

**Figure 9.**
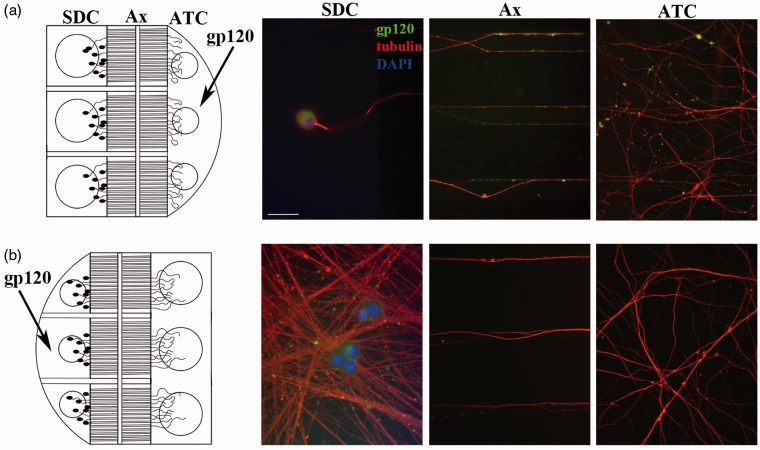
Internalized gp120 undergoes retrograde but not anterograde transport along DRG axons. Primary DRG neurons were grown in compartmentalized microfluidic devices. To examine retrograde transport of gp120, DRG neurons were seeded in the larger compartment to isolate cell bodies fluidically (schematic on a). The ATC was treated with 70 nM gp120 for 4 hr, and then the cells were fixed and stained with an anti-tubulin antibody (in red). Scale bar: 15 µm. Representative images of DRG neurons show that, after 4 hr of treatment, gp120 is present within axons in the microfluidic channels and in cell bodies within the SDC, demonstrating that gp120 has been transported from the ATC to the SDC. Conversely, DRG neurons were seeded in the smaller compartment to isolate their axons fluidically (schematic on b). The SDC was treated with 70 nM gp120 for 4 hr, and then the cells were fixed and stained as in (a). Representative images of DRG neurons show that fluorescein-gp120 was not detected in the microfluidic channels or in the ATC, indicating that gp120 is not transported in the anterograde direction from the SDC to the ATC.

Primary DRG neurons were grown in the SDC compartment with a larger volume of media in the SDC to isolate the cell bodies and dendrites fluidically from the ATC. After this, 70 nM fluorescein-gp120 was added to the ATC of mature DRG neurons for 4 hr (Figure 9(a), diagram on left). Neurons were then fixed and stained with the DM1A antibody. Gp120 in the ATC, microfluidic channels, and SDC was evaluated by fluorescence microscopy. As shown in Figure 9(a), fluorescein-gp120 was found at the ATC, along axons in the microfluidic channels, and within cell bodies in the SDC, thus demonstrating that gp120 had been internalized and retrogradely transported along axons into neuronal cell bodies. Further, the time course of the experiment indicates that gp120 was transported by fast, and not slow, axonal transport, a microtubule motor-dependent process (Morfini et al., 2012). Next, anterograde transport was examined by growing DRG neurons in devices in which the ATC was maintained with a larger volume of media than the SDC to fluidically isolate the ATC (Figure 9(b), diagram on left). The SDC was treated with 70 nM fluorescein-gp120 for 4 hr. As shown in Figure 9(b), fluorescent microscopy demonstrated that the microfluidic channels and ATC lacked fluorescein-gp120, indicating that recombinant gp120 does not undergo anterograde axonal transport in DRG neurons.

## Discussion

Combination antiretroviral treatment has dramatically improved HIV patient mortality and decreased a number of HIV complications (Greene, 2007; Clifford, 2008). However, the prevalence of DSP continues unabated (Ghosh et al., 2012), making this an important disease complication that still requires treatment. Because HIV does not productively infect neurons (An,Groves, Giometto, Beckett, & Scaravilli, 1999), the mechanism for HIV-induced peripheral nerve damage is believed to involve indirect mechanisms. These include neurotoxicity by secretion of inflammatory factors, cytokines, and shed viral proteins such as gp120 (Kaul et al., 2005). The persistence of DSP despite tight viral control by combination antiretroviral therapy may be due to latently infected cells that produce and release viral proteins (Nath, 2002) or low levels of HIV replication leading to gp120 shedding from HIV particles or from infected macrophages (Kranick and Nath, 2012). Gp120 binds to its coreceptor CXCR4 at the plasma membrane of DRG neurons leading to activation of downstream signaling cascades (Apostolski et al., 1993). One proposal is that aberrant activation of this pathway might produce the pain hypersensitivity (Oh et al., 2001; Bhangoo et al., 2009), axonal degeneration, and apoptosis (Melli et al., 2006) characteristic of DSP. However, recent experiments demonstrating gp120 internalization by certain CNS neurons raised the possibility that intracellular gp120 might contribute to DSP pathogenesis (Bachis et al., 2003, 2006). However, whether sensory neurons affected in DSP are capable of internalizing gp120 and how internalized gp120 might contribute to neurotoxicity was unclear.

Here we demonstrate that both F11 cells and primary DRG rat neurons internalized gp120 in a time-dependent manner. Quantitative imaging showed accumulation of gp120 in the perinuclear region over time that began to level off after 2 hr of incubation with recombinant gp120 in the culture media. The highly punctate nature of intracellular gp120-derived fluorescence was consistent with that reported for other cell types (Cefai et al., 1992), including CNS neurons (Bachis et al., 2003). Additionally, heat inactivation and AMD pretreatment experiments demonstrated that gp120 internalization was specific to its biological activity and dependent upon native protein conformation.

Pharmacological experiments with the inhibitor AMD indicated that gp120 internalization was partially dependent on binding to its coreceptor CXCR4 but did not reveal the pathway for internalization. Extracellular gp120 was previously reported to cause neurotoxicity through aberrant activation of CXCR4-linked signaling cascades (Oh et al., 2001; Trushin et al., 2007). However, our results show that gp120 can be internalized, raising the possibility that intra-neuronal gp120 contributes to sensory neuron toxicity. Binding of gp120 to CXCR4 might represent an initial step for gp120 internalization.

Colocalization studies with markers of different endosomal pathways helped define pathways for gp120 internalization. Lack of colocalization with EEA1 and LAMP2 demonstrated that gp120 is not significantly internalized through the common endolysosomal pathway. Rather, colocalization analysis of gp120 with cholera toxin B and with dextran indicated that the bulk of extracellular gp120 was internalized through lipid rafts, with a minor fraction being internalized through fluid phase pinocytosis. Accordingly, disrupting lipid rafts through CD treatment greatly decreased the amount of internalized gp120. This observation was consistent with previous reports showing that clustering of gp120 with its coreceptors within lipid rafts is crucial for HIV infection (Manes et al., 2000; Liao et al., 2001; Popik et al., 2002). Moreover, gp120 was reported to induce CXCR4 movement into lipid rafts for HIV entry (Kamiyama et al., 2009). Based on these results, the major pathway for internalization of gp120 appears to require binding to CXCR4 in association with lipid rafts. Gp120 has been reported to cointernalize with CXCR4 in T-cells (Misse et al., 1999), but internalized CXCR4 reportedly localizes to recycling endosomes in T-cells (Zhang et al., 2004). We do not see colocalization of internalized gp120 with endosomal markers, raising the possibility that sensory neurons and hematopoetic cells utilize different pathways to internalize CXCR4.

The bulk of gp120 was internalized through lipid rafts in neurons and neuron-like cells, but a small fraction was internalized through fluid phase pinocytosis. One possibility is that the fraction of gp120 taken up by fluid phase pinocytosis was due to the nonselectivity of the process (Lim and Gleeson, 2011); in fact, fluid phase pinocytosis is increased if the concentration of a fluorescent ligand is high enough to saturate a receptor-mediated process (Swanson, 1989). However, these two mechanisms have been found concurrently in the internalization of HIV in brain microvascular endothelial cells (Liu et al., 2002), demonstrating that both lipid raft internalization and fluid phase pinocytosis might mediate soluble (shed) gp120 or HIV virion internalization in multiple cell types. Additional experiments are needed to understand whether one or both routes of gp120 internalization contribute to pathogenesis. However, our data suggest that inhibition of lipid raft internalization (through cholesterol-lowering drugs such as statins) or fluid phase pinocytosis (through sodium proton exchange inhibitors like dimethyl amiloride) might have beneficial effects to halt or prevent DSP progression.

What is the fate of internalized gp120 in DRG neurons? If internalization occurs within axons, then gp120 would not be immediately degraded, as degradation of endocytosed protein by lysosomes occurs in the perinuclear region of the neuron (Parton et al., 1992; Tai and Schuman, 2008). Significantly, gp120 was transported in the retrograde direction from isolated DRG axons to their fluidically isolated cell bodies. Retrograde axonal transport of gp120 is consistent with time course studies in F11 cells, as gp120-derived fluorescence first appeared within neurites, gradually accumulating at the perinuclear region at later time points. Although prelysosomal endosomes are among the organelles moving with retrograde axonal transport, colocalization studies with the lysosomal marker LAMP2 rule out a lysosomal destination for gp120, suggesting a different organelle such as a signaling endosome (Howe and Mobley, 2004) or another less well-characterized membrane-bounded organelle. Experiments with microfluidic devices indicated that gp120 was not transported in the anterograde direction from the cell body to axons, consistent with prior work which reported that selective retrograde transport of viruses and viral proteins (Leopold and Pfister, 2006; Berth et al., 2009). Identification of functional roles for the gp120 target organelle is critical.

The present work establishes internalization of gp120 by sensory neurons and characterizes candidate pathways for internalization and intracellular location of gp120. To date, the majority of research examining direct gp120 neurotoxicity in DSP has focused on its binding to CXCR4 and subsequent activation of signaling cascades (Kamerman et al., 2012). Significantly, gp120 follows a pathway by which HIV can enter cells and escape into the cytoplasm to infect cells. As a result, intracellular actions of gp120 that might underlie the toxic effect of gp120 on DRG neurons remain to be characterized. Intracellular actions of gp120 may play a role in the development of dying back neurodegeneration. For example, intracellular gp120 may affect signaling pathways involved in fast axonal transport regulation, which are critical for maintenance of axonal connectivity and neuronal survival (Berth et al., 2009; Morfini et al., 2009). Identifying intracellular effects of this protein within axons may provide important information on the molecular basis for DSP in long-term HIV patients and could lead to the development of novel therapeutic treatments for HIV-related DSP.

## Summary

The neurotoxic HIV glycoprotein gp120 was found to be internalized through lipid rafts and fluid phase pinocytosis and retrogradely transported along axons, providing evidence for intracellular interactions by gp120 in the peripheral nervous system.
